# Confined Concrete in Fiber-Reinforced Polymer Partially Wrapped Square Columns: Axial Compressive Behavior and Strain Distributions by a Particle Image Velocimetry Sensing Technique

**DOI:** 10.3390/s18124118

**Published:** 2018-11-23

**Authors:** Yong-Chang Guo, Shu-Hua Xiao, Jun-Wei Luo, Yu-Yi Ye, Jun-Jie Zeng

**Affiliations:** 1 School of Civil and Transportation Engineering, Guangdong University of Technology, Guangzhou 510006, China; guoyc@gdut.edu.cn (Y.-C.G.); 2111709023@mail2.gdut.edu.cn (S.-H.X.); 2111509050@mail2.gdut.edu.cn (J.-W.L.); 3215002883@mail2.gdut.edu.cn (Y.-Y.Y.); 2 Department of Civil and Environmental Engineering, The Hong Kong Polytechnic University, Hong Kong 999077, China

**Keywords:** FRP, square column, confinement, partial wrapping, FRP-confined concrete, PIV sensing, design-oriented stress–strain model

## Abstract

Strengthening existing reinforced concrete (RC) columns using a partial wrapping strengthening technique (PWST) by fiber-reinforced polymer (FRP) strips has been widely implemented. However, compared with the confinement mechanism of confined concrete in columns strengthened with the FRP full wrapping strengthening technique (FWST), the confinement mechanism of confined concrete in FRP partially wrapped columns is less understood. This paper presents the results of an experimental investigation into the behavior of confined concrete in FRP partially wrapped square columns under axial compression. The effects of FRP strip width and thickness on stress–strain behavior were thoroughly investigated. The novel particle image velocimetry (PIV) non-contact strain sensing technique was adopted to measure the strain in the specimens. Results show that the axial strains as well as the hoop strains are generally larger at the mid-plane of adjacent FRP strips than those at the mid-plane of each FRP strip, and considerable variation in hoop strains along the height of the specimens was observed. Comparisons between the experimental results and predictions by existing design-oriented stress–strain models were carried out to examine the accuracy of the models. A new design-oriented stress–strain model is proposed for confined concrete in FRP partially wrapped square columns and the comparisons between laboratory results and predictions from the proposed model show that the proposed model is superior to the existing models.

## 1. Introduction

**Figure 1:**
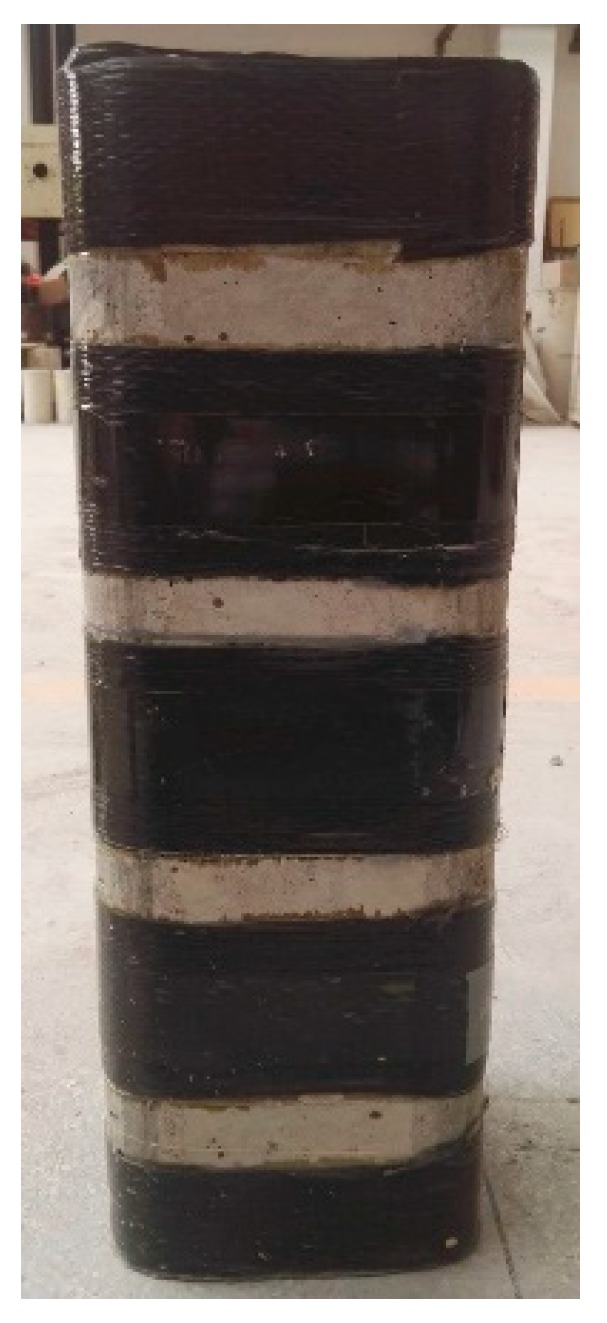
Schematic of the fiber-reinforced polymer (FRP) partial wrapping strengthening technique.

Fiber-reinforced polymer (FRP) composites have been widely used in construction (e.g., [1,2,3]) and one of the most popular utilizations of FRP composites in structural engineering is strengthening existing reinforced concrete (RC) columns using the FRP full wrapping strengthening technique (abbreviated as FWST henceforth). Many experimental and theoretical studies have been carried out on confined concrete in FRP fully wrapped columns and it has been found that the strength and axial deformation capacities of concrete are substantially enhanced owing to the FRP confinement (e.g., [4,5,6,7,8,9,10,11,12,13]). For another perspective, RC columns can be strengthened/rehabilitated through the FRP partial wrapping strengthening technique (abbreviated as PWST henceforth) which has recently also been found to be efficient at enhancing the strength as well as the ultimate strain of concrete (e.g., [14,15,16]). PWST is particularly preferred if FWST is not easy to be applied, such as in strengthening columns which have a fewer number of joints. Another advantage of the PWST is that the FRP strip can be applied between two adjacent steel ties of an existing RC column, which avoids the repeated confinement from the steel ties if the FWST was adopted. In strengthening RC columns using the PWST, columns are generally wrapped with FRP strips/hoops (Figure 1) rather than wrapping columns with longitudinally continuous FRP jackets and the confined concrete is referred to as FRP ring-confined concrete or confined concrete in FRP partially wrapped columns hereafter. Several studies have been conducted on confined concrete in FRP partially wrapped circular columns, which demonstrate substantial enhancements in both strength and ductility of concrete in FRP partially wrapped circular columns (e.g., [17,18,19,20,21,22,23,24]).

**Figure 2:**
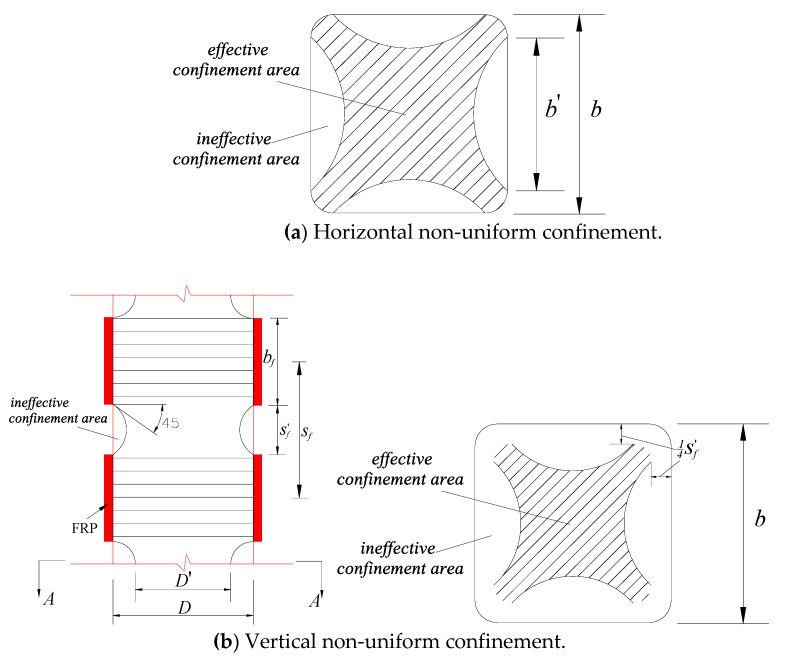
Non-uniform confinement in square columns partially wrapped with FRP strips.

In practical engineering, however, square columns are more commonly seen in industry because of the ease in fabrication of formworks and architectural reasons (e.g., the square cross-sectional shape is generally selected by the architects or the owners), while the confinement mechanism of FRP-confined concrete in square columns is different from that in circular columns. When it comes to confined concrete in square columns, however, the efficiency of confinement is much worse than that of circular columns. The reason for the reduced confinement efficiency is that the confinement in square columns is non-uniform due to the existence of the flat edges and the acute corners in square columns [25,26]. This reduced confinement effect is referred to as horizontal non-uniform confinement in this paper and the reduced confinement effect resulting from the horizontal non-uniform confinement is generally considered using effective confinement area (Figure 2a) based on the well-known “arching action” hypothesis [27,28]. Note that the “arching action” hypothesis has also been used for FRP bar reinforced concrete columns [2,12]. Similarly, for FRP partially wrapped square columns, the confining stresses between two adjacent strips is non-uniform (referred to as vertical non-uniform confinement hereafter, see Figure 2b) and the reduction in confinement between strips is also accounted for by the “arching action”; while at each level in square columns, it is assumed that the area included in the four second-degree parabolas is the effective confinement area [28,29]. Note that the clear spacings between every two adjacent FRP strips ($s_{f}^{\prime }$) are generally identical (Figure 2b). The Italian code [30] suggests using a confinement efficiency factor considering both horizontal non-uniform confinement and vertical non-uniform confinement in PWST. Although some studies have been conducted to evaluate the reliability of the confinement efficiency factor for non-uniformly confined concrete (e.g., [16]), most research has concentrated on PWST for circular columns rather than square columns (e.g., [23,24]), while the FRP confinement efficiency factor based on the “arching action” hypothesis for confined concrete in FRP partially wrapped square columns has never been verified or assessed. Additionally, the axial and hoop strain distributions along the height of the specimens have never been studied. The novel particle image velocimetry (PIV) non-contact strain sensing technique is an appropriate technique for measuring the strain distributions.

The extensive applications of the PWST require a reliable and precise stress–strain model for confined concrete in FRP partially wrapped square columns. It is hitherto well known that confined concrete in FRP fully wrapped square columns exhibits a stress–strain behavior with a first parabolic curve and a second linear curve (e.g., [31,32]) and a number of design-oriented stress–strain models (abbreviated as stress–strain models hereafter) have been proposed for confined concrete in FRP fully wrapped square columns (e.g., [31,33,34]). As mentioned earlier, to bridge the gap between PWST and FWST, a confinement effectiveness factor has been specified for use in stress–strain models by the Italian FRP code [30] and the European FRP code [35] for PWST. However, previous research has been limited to examinations of the precision of the existing stress–strain models (with the confinement effectiveness factor being incorporated) for partially wrapped circular columns (e.g., [16,26]), rather than confined concrete partially wrapped square columns. Consequently, new experimental programs consisting of square columns need to be conducted and the accuracy of the existing stress–strain models for confined concrete in FRP partially wrapped square columns needs to be carefully examined.

To this end, an experimental investigation on the behavior of confined concrete in FRP partially wrapped square columns under axial compression is carried out in the current paper. The main test variables investigated in the experimental study included FRP strip width and thickness. The effects of the width and thickness of FRP strips on the behavior of confined concrete in FRP partially wrapped square columns were illustrated and elaborated. Four typical stress–strain models were then verified by the test results presented in the current paper. A refined stress–strain model was proposed and verified against the test results.

## 2. Experimental Study

### 2.1. Columnar Specimens

The main parameters investigated are the FRP strip width and the FRP strip thickness. The variation of the above parameters leads to a total of 17 middle-scale square columnar specimens tested in the present study. The columnar specimens were produced to have three different FRP thicknesses (i.e., 0.167 mm, 0.334 mm and 0.501 mm) and several different FRP strip widths (including three fully wrapped specimens). The detailed descriptions of the specimens (including the FRP clear spacing ($s_{f}^{\prime }$), the FRP strip width ($b_{f}$), the FRP jacket thickness ($t_{f}$), FRP volumetric ratio ($\rho_{f}$), FRP confinement efficiency ratio ($\rho_{f}k_{v}$) and the unconfined concrete properties ($f_{co}^{\prime }$ and $\varepsilon_{co}$)) are given in Table 1. All the square columnar specimens had a height of 500 mm and a section edge width of 200 mm. The column corner radius is 30 mm, complying with the Chinese Code (GB 50608 2010) [36].

As seen in Table 1, the specimens are classified into five groups (i.e., Group I, II, III, IV and V). Among these specimens, the FRP volumetric ratios ($\rho_{f}$) of specimens F-2, P-3-40-20, P-3-80-40 and P-3-120-60 are identical. The FRP confinement efficiency ratio ($\rho_{f}k_{v}$) of the specimens P-3-30-20, P-3-80-40 and P-3-178-60 are also identical. The FRP volumetric ratio for partially wrapped square columns can be obtained by $\rho_{f}=4t_{f}b_{f}/s_{f}b$. As mentioned earlier, the factor considering the reduction of vertical confinement is the vertical confinement effectiveness factor $k_{v}={\left(1-s_{f}^{\prime }/2b\right)}^{2}$ (*fib* 2001 [35]), where b is the width of the square columns and $s_{f}^{\prime }$ is the clear spacing (i.e., clear spacing between two adjacent FRP strips). Consequently, the specimens with an identical parameter can be easily compared with each other to assess the confinement efficiency factor specified in the current design codes.

Each column has a specified name (see Table 1) with the letter “P” to denote “partial” (“F” represents “full”) at the beginning and then followed by a value denoting the FRP layer numbers. Following this, there is a number denoting the width of the strips and at the very end, there is a number denoting the clear spacing. For example, ‘P-3-100-40’ represents a specimen wrapped with three-layer FRP strips with a width of 100 mm and a clear spacing of 40 mm.

### 2.2. Material Properties

The mix proportion of concrete is given in Table 2 and the concrete was casted into wooden molds with required sizes. The same batch of concrete was used to cast the specimens and the properties of unconfined concrete were obtained from a compression test (ASTM C469 2002 [37]) on the unconfined square column (R-0) (see Table 1). The fibers in the FRP strip were oriented in the hoop direction and a 150 mm overlapping zone was adopted for all the wrapped specimens. Unlike the bond between the FRP and the concrete in a beam or beam–column member in which the bond strength is significant (e.g., [38,39,40]), the failure of FRP will not occur in the overlapping zone (i.e., debonding failure generally will not appear) and the FRP serves as a confining material in a column member. That is to say, the bond strength between the layers of FRP, rather than that between the concrete and FRP, is critical to the confinement effectiveness. Note that the columns should be wrapped with FRP rings over the whole height in practice.

The material properties of FRP were determined by FRP flat coupon tensile tests, following ASTM D3039 2008 [41]. The width and length of the carbon FRP (CFRP) flat coupons were 25 mm and 250 mm, respectively. Tensile test results were useful only when the coupons failed by FRP rupture near the middle of the specimen. The tensile rupture strain ($\varepsilon_{f}$), tensile strength ($f_{f}$) and tensile elastic modulus ($E_{f}$) are 0.0191, 4308.6 MPa and 227.3 GPa, respectively (Table 3).

### 2.3. Test Setup 

**Figure 3:**
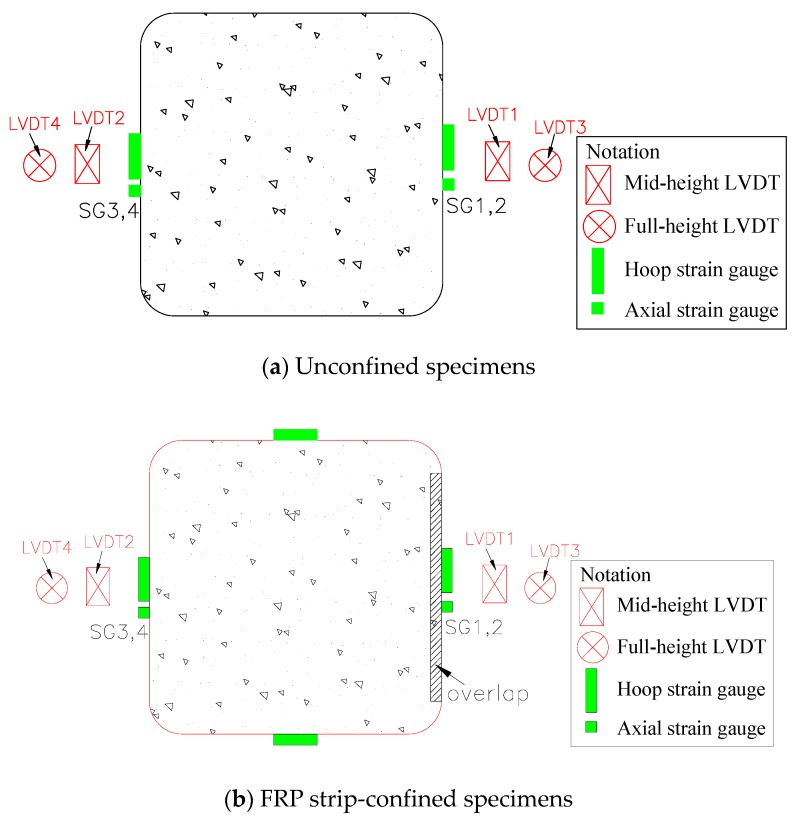
Instrumentations. LVDT = linear variable displacement transducer. SG = strain gauge.

The mid-height axial deformation of the columnar specimens was recorded by two linear variable displacement transducers (LVDTs) (LVDT 1 and 2, see Figure 3), covering a gauge length of 200 mm. Another two LVDTs (LVDT 3 and LVDT 4, see Figure 3) were included to measure the full-height axial shortening. Four unidirectional strain gauges (SGs) were installed at the mid-height of the unconfined columnar specimens to measure the strains in the concrete; among them, two 100 mm SGs at 180° apart were for axial strains and the other two 80 mm SGs 180° apart were for lateral strains. The hoop and axial strains in the FRP jacket at the mid-height plane were captured by six unidirectional SGs mounted on the mid-height plane of each FRP-wrapped columnar specimen (see Figure 3b for more details).

**Figure 4:**
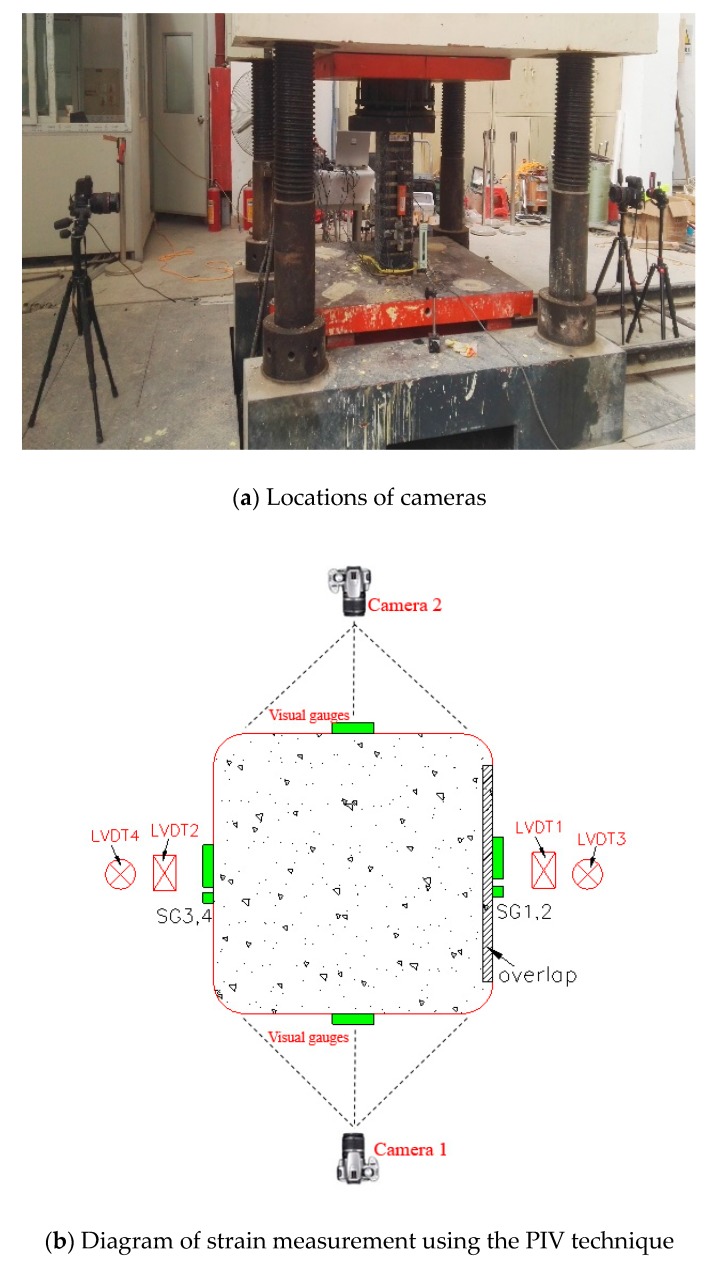
Strain measurement using the particle image velocimetry (PIV) sensing technique.

**Figure 5:**
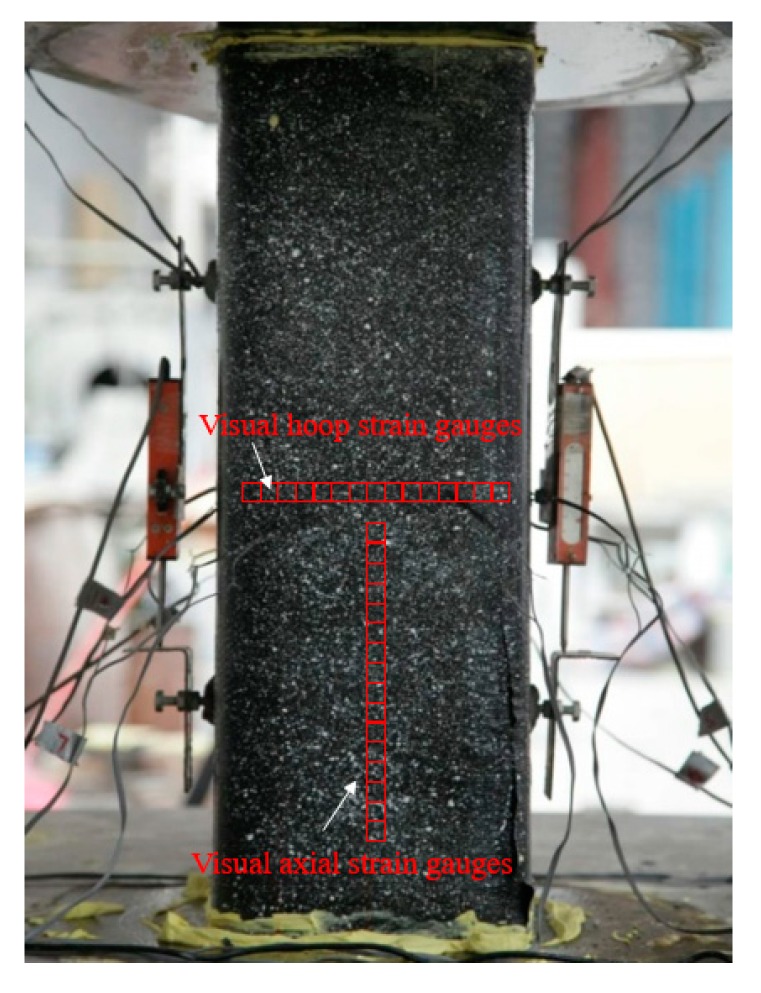
The visual strain gauges based on PIV image acquisition.

Additionally, the PIV sensing technique was employed to record the strains (i.e., both axial and hoop strains) along the height of selected FRP strip-confined specimens (whether the technique was applied is indicated in Table 1). This PIV sensing technique was realized by tracking the spatial variation of brightness of the test patches utilizing the geoPIV software developed by White et al. [42]. An image analysis algorithm in the software is adopted to track the displacement of each patch in the subsequent images. The focused surfaces of the specimens were sprayed with black paint while the texture was formed by white paint. Details of the test set-up can be found in Zeng et al. [22]. Two Canon 5D-Mark II digital cameras were utilized to record the images of the displacement of the specimens during testing (Figure 4a). The interval between two successive shutters was three seconds. The center normal vector of the lens is perpendicular to the vertical shaft of the columns, while the schematic diagrams in Figure 4b show the locations of the cameras. Figure 5 shows the locations of visual strain gauges based on the PIV sensing acquisition. Note that the PIV technique has been employed to measure the strains in FRP (e.g., [22,43]) and the accuracy of the PIV sensing technique has been proved by Zeng et al. [22] by applying both the PIV sensing technique and conventional strain measurement techniques to the same specimen. The stress–strain curves of the confined concrete from the both sources were close to each other.

High-strength gypsum mortar was used to level each of the specimen ends, and thus the axial load is uniformly applied on the whole section. A column compression test was realized in a hydraulic machine for compression loading using a displacement control mode with a 0.24 mm/min rate for unconfined columnar specimens and a 0.50 mm/min rate for FRP strip-confined columnar specimens. All test data was recorded by data logger.

## 3. Results and Discussions

### 3.1. Failure Modes

**Figure 6:**
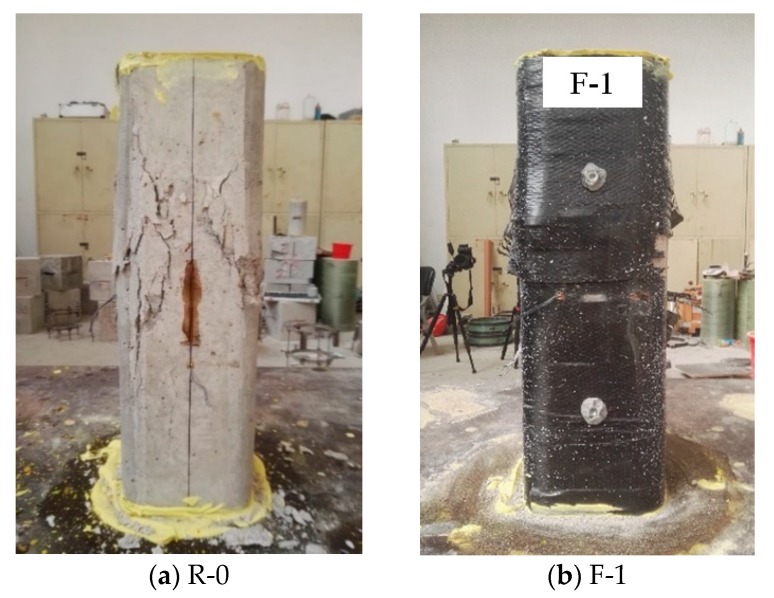

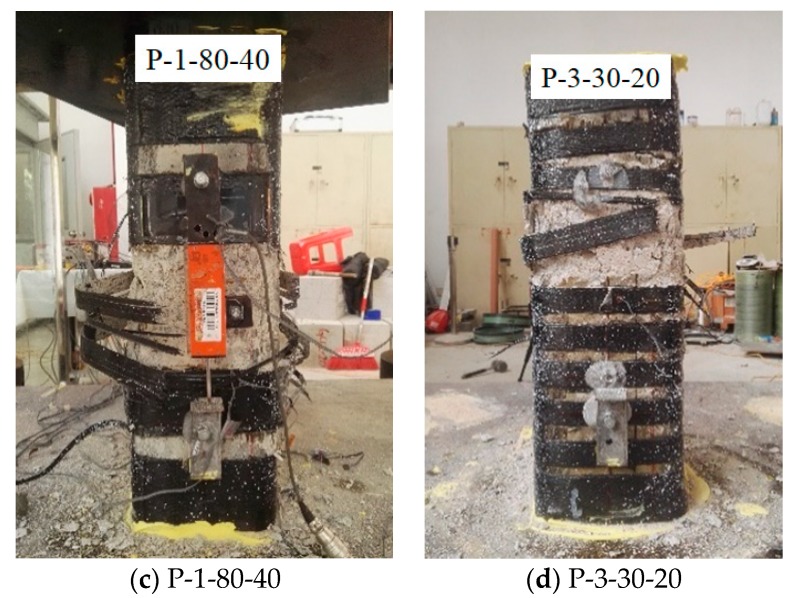
Typical failure modes of selected specimens.

The failure models of the chosen columns, as typical examples, are shown in Figure 6. Obviously, the unconfined specimen failed by concrete crushing failure with numbers of inclined cracks in the mid-height (Figure 6a). Square columns with FRP full wrapping failed by FRP rupture at the mid-height (Figure 6b), which is in good agreement with previous studies (e.g., [26]). The FRP partially wrapped specimens generally underwent FRP rupture failure near (or at) the mid-height, and close to the corner–flat side transition points, as shown in Figure 6c,d. Cracks in the concrete appeared at the clear spacing at an earlier loading stage, and then the cracks expanded gradually until FRP rupture took place, accompanied by concrete crushing failure. The FRP strips at both ends of the columnar specimens were shown to be undamaged when the failure occurred, which is most likely due to the additional confinement from the loading pedestals of the machine [44].

### 3.2. Stress–Strain Responses

**Figure 7:**
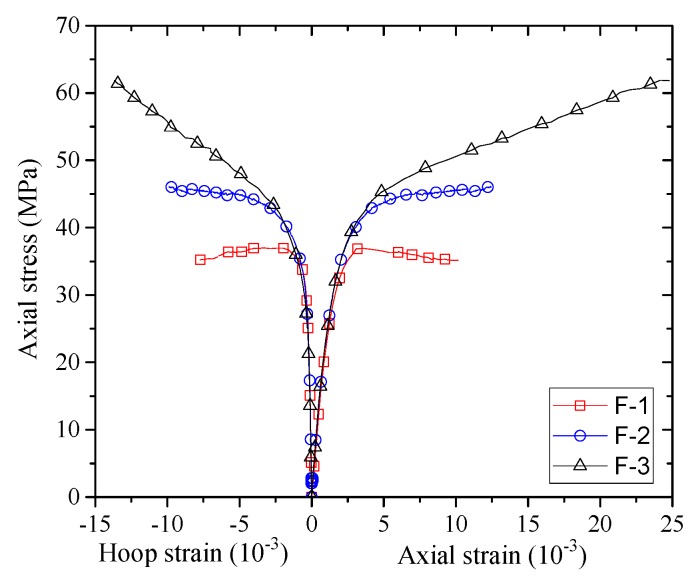
Stress–strain curves of concrete in the Group I specimens.

**Figure 8:**
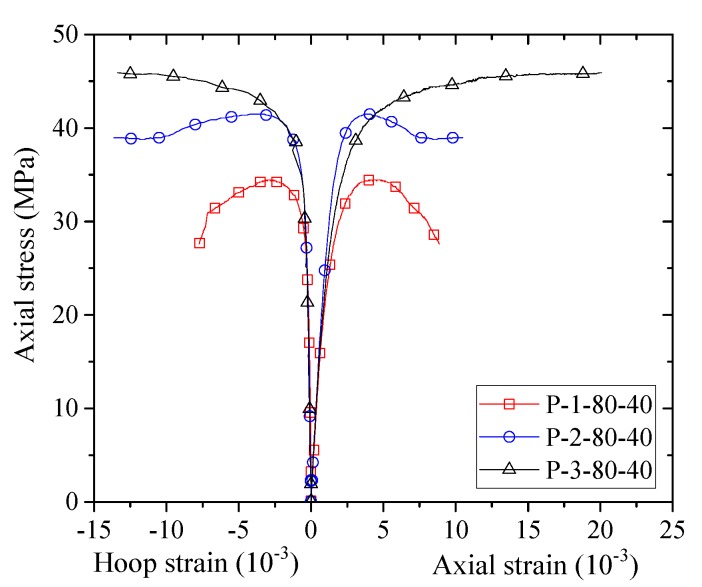
Stress–strain curves of concrete in the Group II specimens.

**Figure 9:**
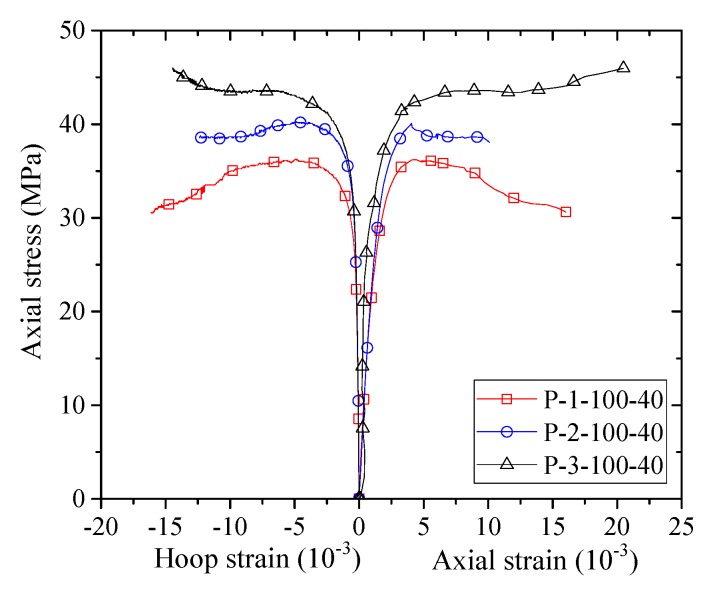
Stress–strain curves of concrete in the Group III specimens.

**Figure 10:**
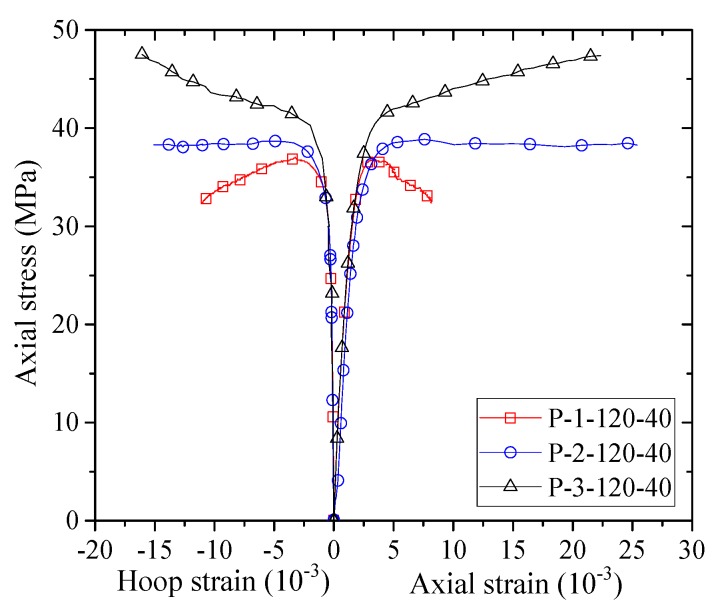
Stress–strain curves of concrete in the Group IV specimens.

**Figure 11:**
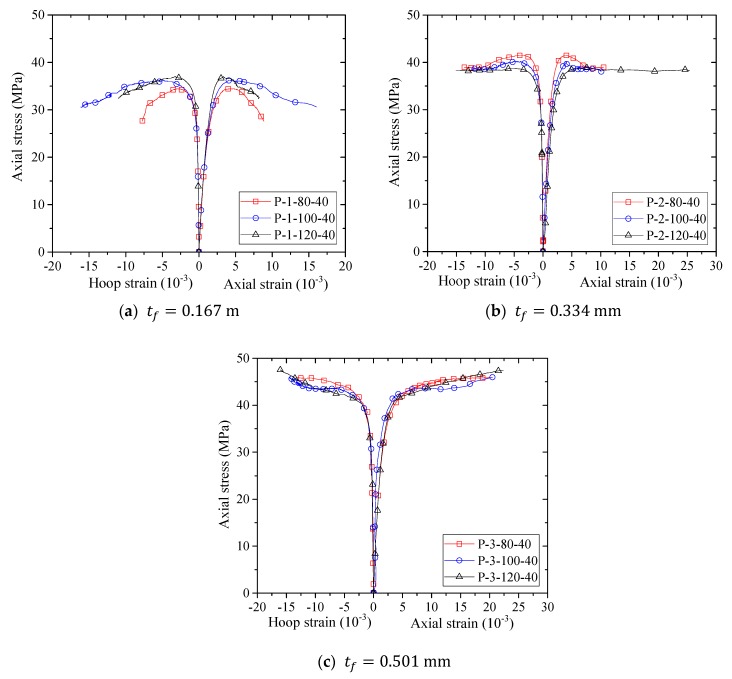
Stress–strain curves of concrete in specimens with different FRP strip thicknesses.

The stress–strain curves of confined concrete in the columnar specimens are shown in Figure 7, Figure 8, Figure 9, Figure 10 and Figure 11. As the axial stresses in a confined square column are non-uniform, the presented axial stresses in concrete are average stresses which were obtained by dividing the axial loads by the gross sectional area. The average mid-height LVDT readings were adopted for the axial strains, which have also been adopted by previous researchers (e.g., [45]). The reported stress–strain curves cease when FRP rupture took place. The stress–strain curves of confined concrete in the FRP fully wrapped square columns (Group I) are shown in Figure 7. For specimens F-2 and F-3, the axial stresses show monotonic ascending, while specimen F-1 shows a slightly descending second portion. The reason for the descending behavior is that the column was confined with a small amount of FRP while a monotonic ascending behavior required sufficient FRP confinement. 

Figure 8, Figure 9, Figure 10 and Figure 11 show the stress–strain curves of confined concrete in the FRP partially wrapped columnar specimens. A clear observation from the stress–strain curves is that they exhibit a three-portion behavior with a first linear portion, a second parabolic transition portion and a third linear portion [16]. However, the second transition portion is short, and the stress–strain curves can be approximately represented by the typical two-portion stress–strain curves. The difference of Group II, III and IV specimens is that the FRP strip widths are different; namely, the thicknesses of FRP strips are different in each group, leading to a direct comparison between specimens with an identical FRP strip width. From Figure 8, Figure 9 and Figure 10 we can see that both the peak stress and the slope of the second portion increase with the increase of thickness of the FRP strips. The stress–strain curves of the specimens with the same FRP strip thickness but different FRP strip widths are exhibited in Figure 11. It is interesting to find that the FRP strip width is approximately independent to the ultimate axial stress. The peak stresses are slightly different with the increase in FRP strip width, as can be seen from Figure 11. However, the slight difference between the peak stresses may result from the intrinsic discreteness of concrete and thus gives little implication to the conclusion. This contradicts the findings from previous studies that an increase in the FRP strip width causes an increase in the peak stress of confined concrete (e.g., [16]). One possible reason is that the stress–strain response of concrete in FRP partially wrapped square columns is more susceptible to the clear spacing rather than the width of FRP strips. 

**Figure 12:**
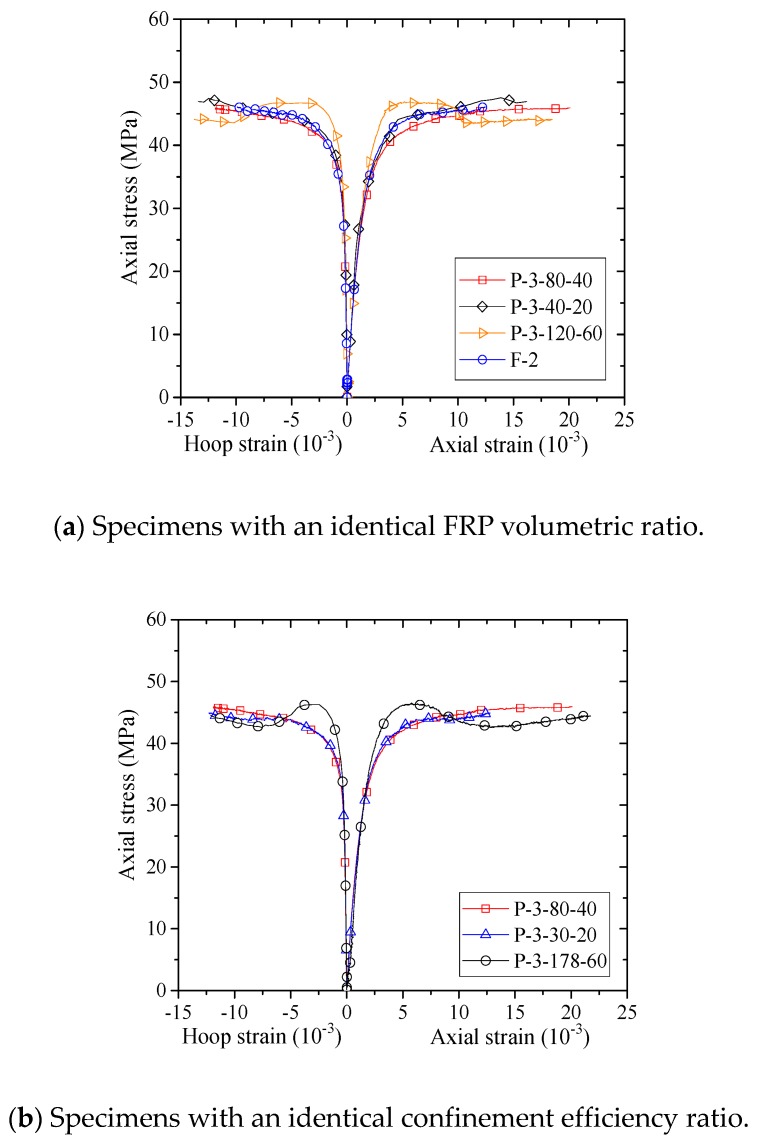
Stress–strain curves of concrete in the specimens with an identical FRP volumetric ratio and an identical confinement efficiency ratio.

The stress–strain responses of the specimens with an identical FRP volumetric ratio ($\rho_{f}$) and the specimens with an identical FRP confinement efficiency ratio ($\rho_{f}k_{v}$) are reported in Figure 12. In this paper, the FRP confinement efficiency ratio is referred to as the product of the FRP volumetric ratio ($\rho_{f}$) and the vertical confinement efficiency factor ($k_{v}$). It can be seen from Figure 12 that for the specimens with an identical $\rho_{f}$, the stress–strain curves are close to one another, indicating the FRP volumetric ratio is a vital factor that dominates the stress–strain response of confined concrete in FRP partially wrapped square columns. A more attentive view of Figure 12a (specimens in this figure have an identical FRP volumetric ratio but different clear FRP strip spacings) sheds light on important evidence showing that the specimen with a narrower clear spacing exhibited a slightly improved stress–strain curve, implying that the stress–strain response of confined concrete is highly related to the FRP strip clear spacing rather than the FRP strip width. More explanation can be found by comparing the stress–strain curves of specimens with an identical $\rho_{f}k_{v}$, as is shown in Figure 12b. For specimens in Figure 12b (i.e., P-3-80-40, P-3-30-20 and P-3-178-60), the FRP confinement efficiency ratio ($\rho_{f}k_{v}$) is identical. The difference between ultimate axial stresses of the specimens with an identical $\rho_{f}k_{v}$ is smaller than the difference between those of the specimens with an identical $\rho_{f}$; it is thus believed that $k_{v}$ does play a more significant role in determining the behavior of confined concrete in FRP partially wrapped square columns than the FRP volumetric ratio.

### 3.3. Ultimate Axial Stress and Strain

The experimental results for the columnar columns are given in Table 1. The ultimate axial stress ($f_{cu}^{\prime }$) equals to the peak axial stress ($f_{cc}^{\prime }$) for specimens with a monotonic ascending stress–strain response, and thus only the peak axial stresses are given in Table 1. Note that for the results from specimens with a descending second portion, the peak axial stresses instead of the ultimate axial stresses are discussed. The normalized peak axial stresses ($f_{cc}^{\prime }/f_{co}^{\prime }$) and normalized ultimate axial strains ($\varepsilon_{cu}/\varepsilon_{co}$) are also reported in Table 1. As shown in Table 1, the normalized peak axial stresses ($f_{cc}^{\prime }/f_{co}^{\prime }$) range from 1.01–1.37 and the normalized ultimate axial strains ($\varepsilon_{cu}/\varepsilon_{co}$) range from 3.00–9.28. $f_{cc}^{\prime }/f_{co}^{\prime }$ and $\varepsilon_{cu}/\varepsilon_{co}$ increase with the increase in FRP strip thickness and width, which can be easily seen from Table 1. It should be mentioned that although the $f_{cc}^{\prime }/f_{co}^{\prime }$ for several specimens is low (e.g., P-1-80-40 and P-1-100-40), the axial strain enhancement ratio is quite high, which is also valuable in practice. For specimens with an identical FRP volumetric ratio, the $\varepsilon_{cu}$ of specimens with partial FRP confinement are generally larger than those of specimens with full FRP confinement, as can be concluded by comparing the ultimate axial strains of specimens F2, P-3-80-40, P-3-40-20 and P-3-120-60.

**Figure 13:**
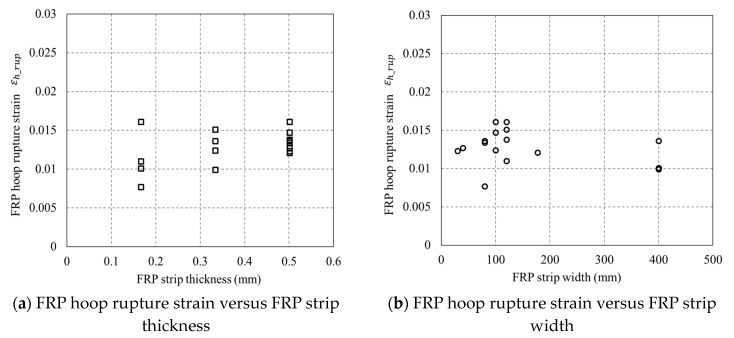
Relationship between FRP hoop rupture strain and FRP strip thickness/width.

Figure 13 shows the FRP hoop rupture strains ($\varepsilon_{h,rup}$) versus the FRP strip thickness/width. FRP hoop rupture strains were found to be independent to the thickness and width of FRP strips. The FRP strain efficiency factor (i.e., the FRP hoop rupture strain over the FRP coupon tensile rupture strain) for each specimen is given in Table 1. The average FRP strain efficiency factor is 0.67, which is very close to the FRP strain efficiency ratios reported in previous studies (e.g., [46,47,48,49]).

### 3.4. Strain Distributions

**Figure 14:**
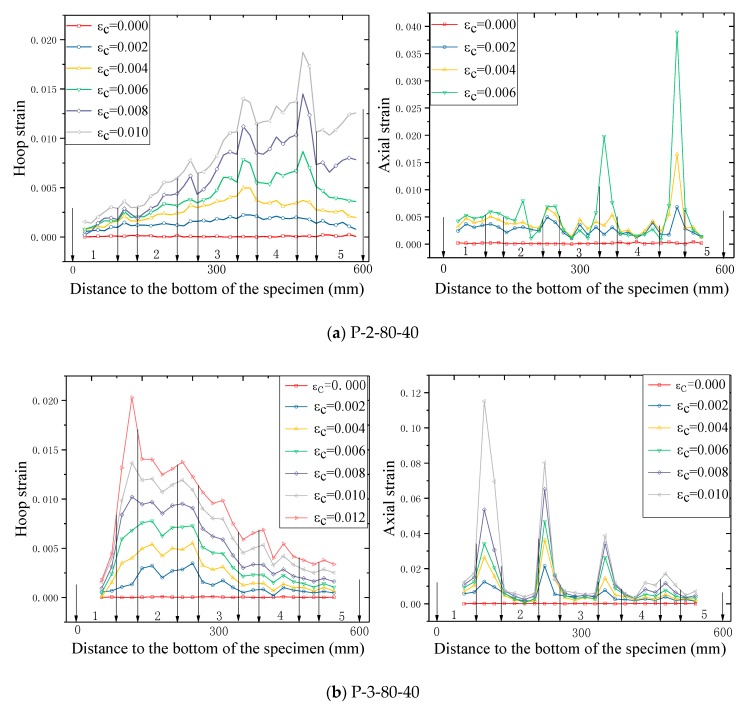

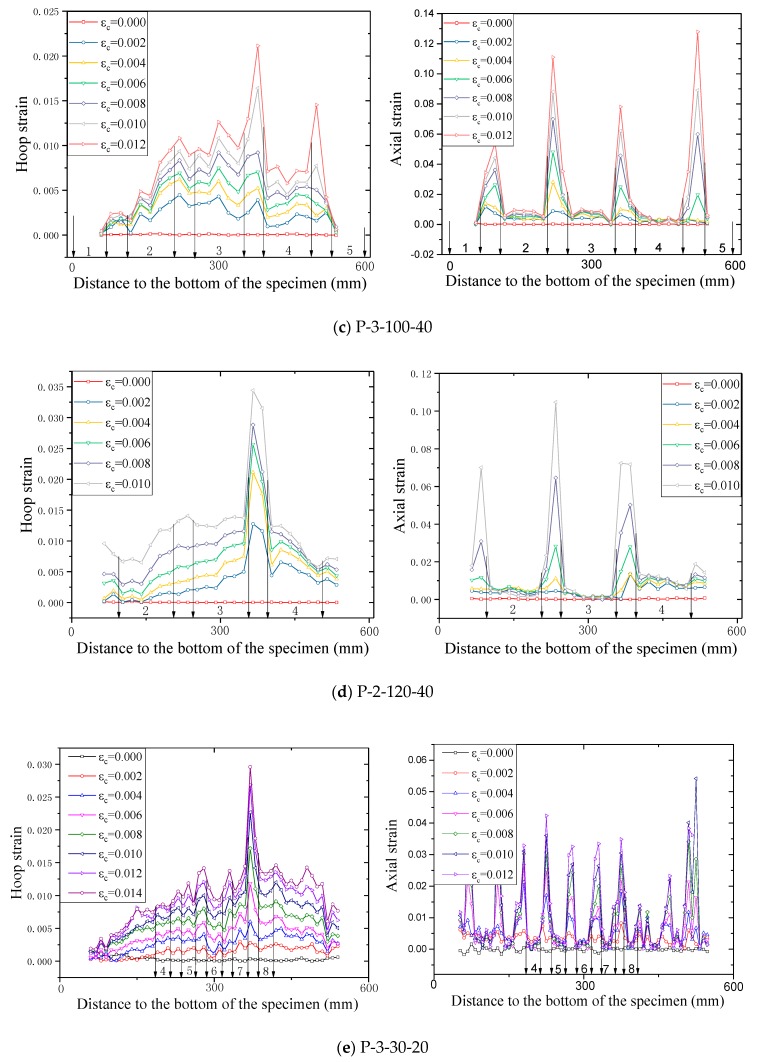

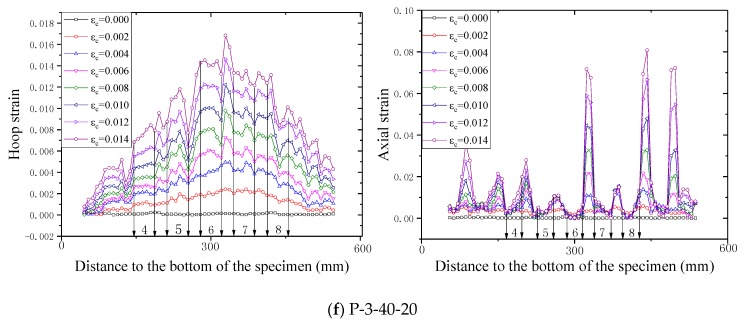
Strain distribution along the specimen height using the PIV sensing technique ($\varepsilon_{c}$ = axial strain of concrete).

The distribution of strains over the specimens at different hoop strain levels is shown in Figure 14, in which the horizontal axis is the distance between the current level to the bottom of the columnar specimen. Note that in Figure 14, the locations of the FRP strips are denoted by the numbers between two adjacent arrows. All the strains reported in Figure 14 were recorded utilizing the PIV sensing technique, with the virtual SGs being shown in Figure 5. The gauge length for all the optical strain gauges in the PIV system was 20 mm, which equals to that of the foil SGs for FRP strains adopted in the current study.

The axial strains increase consistently at all hoop strain levels, suggesting a credence of the PIV sensing technique. It can be seen from Figure 14 that, generally, the axial strains as well as the hoop strains in the concrete between two adjacent FRP strips are larger than those in the FRP (i.e., at the mid-plane of each FRP strip) for the test columnar specimens, which has also been found by Zeng et al. [22] on circular columns. This is because at a given hoop strain, the confinement in the concrete between two adjacent FRP strips is weaker than that in the concrete at the mid-plane of each FRP strip level, leading to a larger strain in the mid-way concrete. This also explains why the $\varepsilon_{cu}$ of confined concrete in FRP partially wrapped square columns is smaller than that of confined concrete in FRP fully wrapped square columns, as has been mentioned in the previous section. It is interesting to find that the strains on specimen P-2-80-40 were concentrated toward the upper end of the specimen while the strains on specimen P-3-80-40, conversely, concentrated toward the bottom end. This is probably due to the local failure of concrete, owing to its homogeneity, which was also reported by Bisby and Take [48].

At the earlier loading stage (i.e., at a lower hoop strain level), FRP hoop strains remained small and were evenly distributed over the specimens. However, they increased rapidly and distributed non-uniformly when the average axial stress of concrete approached the unconfined concrete strength, as can be clearly seen in Figure 14. Subsequently, a vertical asymmetrical FRP hoop strain distribution is observed, although the columnar specimens are vertically symmetrical. Similar results have also been observed by Bisby and Take [48]. The inhomogeneity of concrete may also increase the variation of the FRP hoop strain distribution. The hoop strain variation also helps to explain why sometimes the relationship between hoop strain and axial strain is quite large (e.g., 0.5) and the hoop strains varied in quite a large range as reported in most previous studies on FRP-confined concrete, as well as confined concrete in FRP partially wrapped columns (e.g., [22]).

## 4. Design-Oriented Stress–Strain Models for Confined Concrete in FRP Partially Wrapped Square Columns

### 4.1. Actual FRP Confining Stress in Confined Concrete in FRP Partially Wrapped Square Columns

Sheikh and Uzumeri (1980) [27] proposed to use the “arching action” hypothesis for steel-confined concrete and the effective confinement area has been proposed based on the “arching action” hypothesis [27,28]. Subsequently, the “arching action” hypothesis was adopted by the Italian code [30] and the European code [35] for the design of columns strengthened by PWST. Based on the “arching action” hypothesis, the actual confining stresses in concrete can be found by the following Equation:

(1) $$f_{l,a}=k_{v}k_{s}\sigma_{l}=k_{v}k_{s}\frac{\rho_{f}E_{f}\varepsilon_{h}}{2}$$

where $\rho_{f}$ is the FRP strip volumetric ratio, $\sigma_{l}$ is the confining stress for columns with an FRP volumetric ratio of $\rho_{f}$.

Compared with circular columns, the reduced confinement in square columns can be evaluated using the following shape factor $k_{s}$, according to the “arching action” as well in the horizontal level [30,35,46]:

(2) $$k_{s}=1-\frac{{\left(h-2r_{c}\right)}^{2}+{\left(b-2r_{c}\right)}^{2}}{3A_{g}}$$

**Figure 15:**
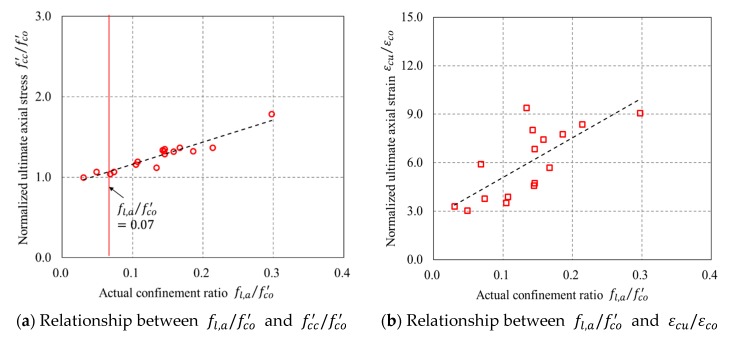
Relationship between actual confinement ratio ($f_{l,a}/f_{co}^{\prime }$) and normalized ultimate axial stress ($f_{cc}^{\prime }/f_{co}^{\prime }$)/normalized ultimate axial strain ($\varepsilon_{cu}/\varepsilon_{co}$ ).

Figure 15a shows the relationship between the actual confinement ratio (i.e., $f_{l,a}/f_{co}^{\prime }$) and $f_{cc}^{\prime }/f_{co}^{\prime }$, which exhibits a linearly proportional relationship between $f_{cc}^{\prime }/f_{co}^{\prime }$ and $f_{l,a}/f_{co}^{\prime }$. Figure 15a also indicates that a $f_{l,a}/f_{co}^{\prime }$ value of greater than 0.07 leads to a strain hardening behavior of confined concrete in FRP partially wrapped square columns, demonstrating that the definition of the minimum amount of FRP derived from FRP-confined circular columns [47,50] is also applicable to FRP-confined square columns. Figure 15b shows a proportional relevance between $\varepsilon_{cu}/\varepsilon_{co}$ and $f_{l,a}/f_{co}^{\prime }$, while it is not as high as that between $f_{cc}^{\prime }/f_{co}^{\prime }$ and $f_{l,a}/f_{co}^{\prime }$.

### 4.2. Existing Stress–Strain Models

Single-portion models (e.g., [51,52]) and two-portion models (e.g., [29,33,53,54]) are two typical types of stress–strain models for concrete confined with FRP [43], both of which have been concluded from previous studies. For single-portion models (e.g., [51]), the following curve [51] is generally employed:

(3) $$\sigma_{c}=E_{2}\varepsilon_{c}+\frac{\left(E_{c}-E_{2}\right)\varepsilon_{c}}{{\left[1+{\left(\frac{E_{c}-E_{2}}{f_{i}}\varepsilon_{c}\right)}^{n}\right]}^{1/n}}$$

where $f_{i}$ is the interception point of the stress axis with the asymptote of the second portion of the stress–strain curve; $\sigma_{c}$ and $\varepsilon_{c}$ are the axial stress and axial strain of confined concrete, respectively; $E_{c}$ is the unconfined concrete elastic modulus; $E_{2}$ is the second portion slope; and n is a shape parameter. 

Cao et al. [54] adopted the following Equation [55] for a single-portion model:

(4) $$\sigma_{c}=\left[\left(E_{c}\varepsilon_{n}-f_{o}\right)e^{-\frac{\varepsilon_{c}}{\varepsilon_{n}}}+f_{o}+E_{2}\varepsilon_{c}\right]\left(1-e^{-\frac{\varepsilon_{c}}{\varepsilon_{n}}}\right)$$

(5) $$\varepsilon_{n}=n_{1}\times f_{o}/E_{c}$$

where $f_{o}$ is the limit stress for the first portion and is assumed to equal to the unconfined concrete strength $f_{co}^{\prime }$, and $n_{1}$ is a parameter which controls the shape of the transition region.

Lam and Teng’s stress–strain model [29], first proposed in an earlier study [47], is a typical two-portion stress–strain model which has been widely adopted for design use (e.g., [36,49,56,57]). Lam and Teng adopted the following Equations:

(6a) $$\sigma_{c}=\{\begin{array}{c} E_{c}\varepsilon_{c}-\frac{{\left(E_{c}-E_{2}\right)}^{2}}{4f_{co}^{\prime }}\varepsilon_{c}^{2}\;\left(0\leq \varepsilon_{c}\leq \varepsilon_{t}\right)\\ f_{co}^{\prime }+E_{2}\varepsilon_{c}\;\left(\varepsilon_{t}\leq \varepsilon_{c}\leq \varepsilon_{cu}\right) \end{array}$$

(6b) $$E_{2}=\frac{f_{cc}^{\prime }-f_{co}^{\prime }}{\varepsilon_{cu}}$$

where $\varepsilon_{t}$ is the transition axial strain determined by the following Equation:

(7) $$\varepsilon_{t}=\frac{2f_{cc}^{\prime }}{E_{c}-E_{2}}$$

Wei and Wu’s [33] stress–strain model, similarly, is also made of a parabolic first segment and a linear second portion:

(8a) $$\sigma_{c}=\{\begin{array}{l} E_{c}\varepsilon_{c}+\frac{f_{t}-E_{c}\varepsilon_{t}}{\varepsilon_{t}^{2}}\varepsilon_{c}^{2}\;\left(0\leq \varepsilon_{c}\leq \varepsilon_{t}\right)\\ f_{t}+E_{2}\left(\varepsilon_{c}-\varepsilon_{t}\right)\;\left(\varepsilon_{t}<\varepsilon_{c}\leq \varepsilon_{cu}\right) \end{array}$$

(8b) $$\varepsilon_{t}=\frac{f_{t}+f_{cu}^{\prime }+E_{c}\varepsilon_{cu}-\sqrt{{\left(f_{t}+f_{cu}^{\prime }+E_{c}\varepsilon_{cu}\right)}^{2}-8f_{t}E_{c}\varepsilon_{cu}}}{2E_{c}}$$

where $E_{2}$ is calculated by Equation (6b) and $f_{t}$ is the transition stress (see Table 4). Note that the two-portion models have separate expressions for the axial stress and the axial strain at transition [33,58,59].

Apart from the above stress–strain models, many other investigations have been focused on only $f_{cc}^{\prime }$ and $\varepsilon_{cu}$ (e.g., [34,51,60,61,62]). To popularize the application of partially FRP-confined concrete and obtain a reliable design, it is of cardinal importance to verify existing models for confined concrete in FRP partially wrapped square columns. Existing research (e.g., [16,47]) has found that Lam and Teng’s model (2003) [29] and Wei and Wu’s model (2012) [33] are superior to the others for confined concrete in FRP fully wrapped square columns. In addition to Lam and Teng’s [29] model and Wei and Wu’s [33] model, two new stress–strain models (Cao et al. 2016 [54]; Guo et al. 2019 [63]) which were proposed recently are also included in the assessment in the current study. The details of the above models are outlined in Table 4.

### 4.3. Comparisons

**Figure 16:**
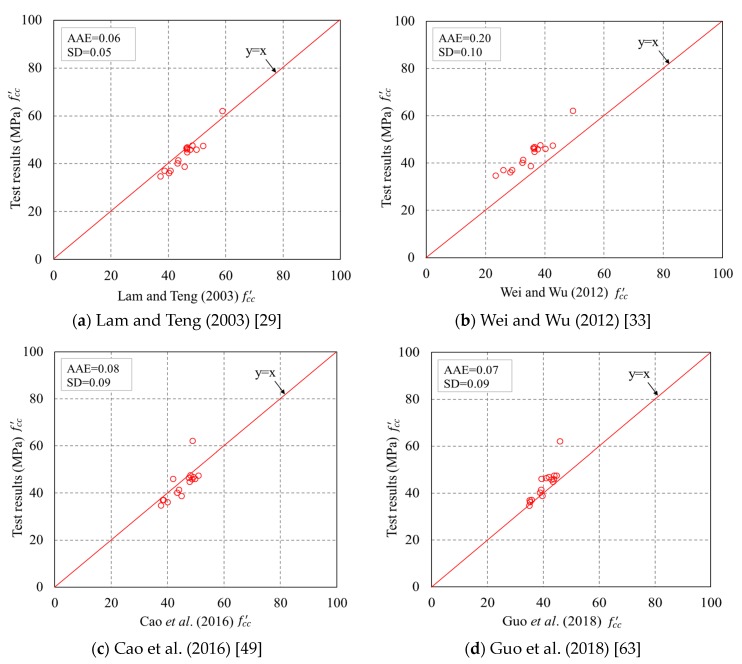
Performance of the existing models in predicting the ultimate axial stresses. AAE = average absolute error. SD = standard deviation.

**Figure 17:**
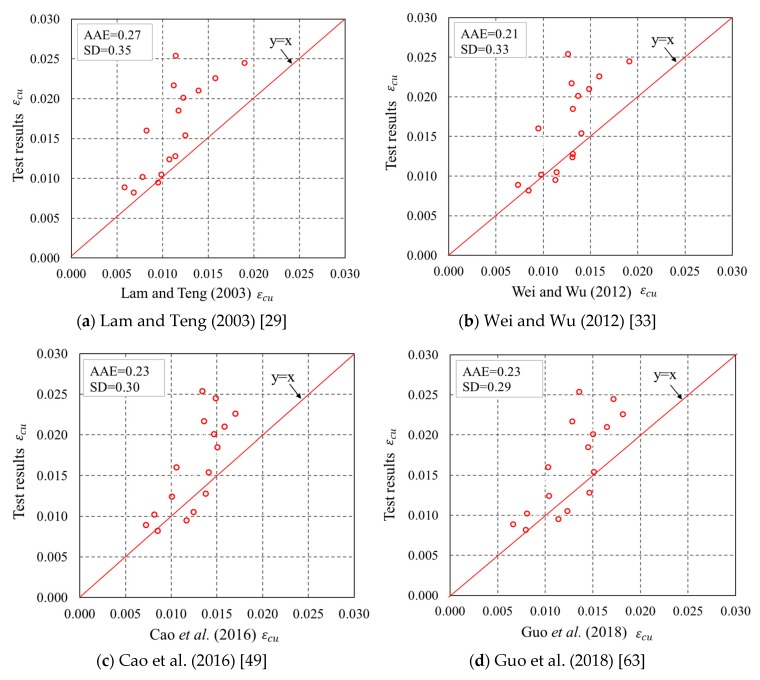
Performance of the existing models in predicting the ultimate axial strains.

The $f_{cc}^{\prime }$ and $\varepsilon_{cu}$ of the specimens predicted by the four representative models were compared with the experimental results. The assessment based on comparisons between experimental and theoretical results of the ultimate condition can properly tell the accuracy of the models as the ultimate condition approximately determines the shape of the stress–strain curve. The predicted curves cease at a hoop strain equal to the average FRP hoop rupture strain. The standard deviation (SD) and average absolute error (AAE) between test results and predictions were utilized to assess the representative models, as can be seen in Figure 16 and Figure 17. It is found that in Figure 16, Lam and Teng’s [29] model is superior to the other three models for $f_{cc}^{\prime }$. For Lam and Teng’s [29] model, both the AAE and SD values are small, indicating a good accuracy of the model in predicting $f_{cc}^{\prime }$ of confined concrete in FRP partially wrapped square columns. The experimental $\varepsilon_{cu}$ are compared with the theoretical results, as shown in Figure 17. An interesting finding from Figure 17 is that Wei and Wu’s [33] and Guo et al.’s [63] models are slightly better than the other models for $\varepsilon_{cu}$. However, the accuracies of all four representative models are similar with respect to $\varepsilon_{cu}$, and both the AAE and SD values are quite large, implying that all four representative models are inaccurate in terms of generating $\varepsilon_{cu}$.

### 4.4. Proposed Stress–Strain Model for Confined Concrete in FRP Partially Wrapped Square Columns

The above discussions demonstrate that none of the four stress–strain models can properly predict the test columns. This paper, consequently, proposes a new stress–strain model for confined concrete in FRP partially wrapped square columns. Based on the model of Teng et al. (2009) [64] for concrete in circular columns fully wrapped with FRP jackets, an improved version of the stress–strain model for confined concrete in FRP partially wrapped circular columns was proposed by Guo et al. [63], in which $f_{cc}^{\prime }$ and $\varepsilon_{cu}$ are calculated by the following new Equations:

(9) $$\frac{f_{cc}^{\prime }}{f_{co}^{\prime }}=a_{1}+a_{2}\left(\rho_{Ke}-a_{3}\right)\rho_{\varepsilon }$$

(10) $$\frac{\varepsilon_{cu}}{\varepsilon_{co}}=b_{1}+b_{2}\rho_{Ke}^{b_{3}}\rho_{\varepsilon }^{b_{4}}$$

in which $a_{1}$, $a_{2}$, $a_{3}$, $b_{1}$, $b_{2}$, $b_{3}$ and $b_{4}$ are constants, $\rho_{Ke}$ is the effective confinement stiffness and $\rho_{\varepsilon }$ is the confinement strain ratio. Note that Equations (9) and (10) have an identical form with the model of Guo et al. [63]. In order to unify the model for both circular and square columns, $a_{1}$, $a_{3}$, $b_{1}$, $b_{3}$ and $b_{4}$ are assumed to be the same as those in Guo et al. (2018) [63], as can be seen in Table 4. The other two factors (i.e., $a_{2}$ and $b_{2}$) are determined very empirically based on limited test results (Teng et al. 2009 [64]) and can be modified by a more representative test database. Therefore, the following values for the constants are proposed: $a_{2}=3.0,\;b_{2}=7.5$.

**Figure 18:**
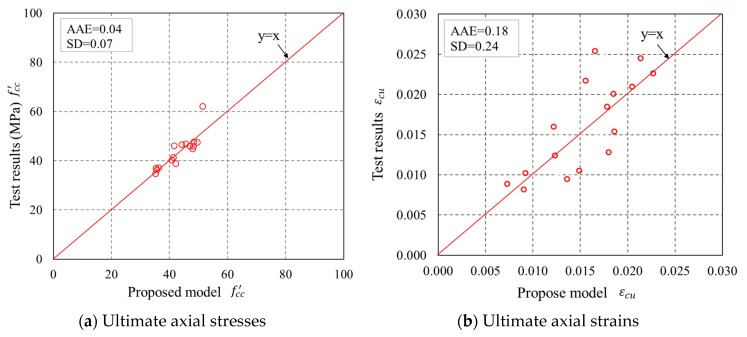
Performance of the proposed model in predicting the ultimate axial stresses and ultimate axial strains.

The accuracy of the new model is examined by comparing the experimental results with the predictions from the proposed model, as shown in Figure 18. It can be seen that the new model is more accurate with respect to both $f_{cc}^{\prime }$ and $\varepsilon_{cu}$. However, the proposed model was based on determination of two constants which is very empirical, and its accuracy is heavily dependent on the volume of the database. Nevertheless, the proposed new model is the first stress–strain model for confined concrete in FRP partially wrapped square columns. The proposed model was only validated by the test results from the current study as the available test results on partially wrapped square columns [23,24] in the literature were based on reinforced concrete columns and the contribution of the internal reinforcing ties made them unsuitable for verification of the proposed model. A more robust and accurate stress–strain model based on adequate experimental results needs to be established in the future for a reliable design of confined concrete in FRP partially wrapped square columns.

## 5. Conclusions

The results of an experimental program consisting of 17 axially loaded square columns have been presented and discussed in this paper. The novel particle image velocimetry (PIV) non-contact strain sensing technique was utilized to measure strains over the specimens. The accuracies of four representative stress–strain models have been examined by the test results presented in the current paper. A new stress–strain model has been proposed and assessed using the test results. The following conclusions can be drawn based on the current study:

(1) The stress–strain responses of confined concrete in FRP partially wrapped square columns exhibit a three-segment behavior with a linear first segment, a parabolic second transition segment and a linear third segment. However, the second transition segment is short, and the stress–strain responses can be approximately represented by the typical two-segment stress–strain curves.
(2) The peak stresses and the slope of the second segment of the stress–strain curves increase with the increase in FRP strip thickness, while the FRP strip width is approximately independent to the ultimate axial stresses and strains of the concrete. The stress–strain response of confined concrete in FRP partially wrapped square columns is highly related to the FRP strip clear spacing rather than the FRP strip width. The FRP hoop rupture strains are independent to the thickness and width of FRP strips.
(3) The difference between ultimate axial stresses of the specimens with an identical FRP confinement efficiency ratio is smaller than the difference between those of the specimens with an identical FRP volumetric ratio, indicating that the vertical confinement effectiveness factor plays a more significant role than the FRP volumetric ratio in influencing the behavior of confined concrete in FRP partially wrapped square columns.
(4) The axial strains as well as hoop strains in the concrete between two adjacent FRP strips are larger than those in the FRP (i.e., at the mid-plane of each FRP strip) for the test columnar specimens and considerable variation is observed in hoop strain readings over the height of the specimens.
(5) Lam and Teng’s (2003) [29] model is superior to the other three models in terms of the ultimate axial stresses. The model of Wei and Wu (2012) [33] and the model of Guo et al. (2019) [63] are slightly better than the other models in predicting the ultimate axial strains, while all four representative models are inaccurate in terms of ultimate axial strains. The proposed model is more accurate in terms of both ultimate axial stress and ultimate axial strains; nevertheless, a more robust and accurate stress–strain model based on adequate experimental results needs to be established.

## Figures and Tables

**Table 1:** Details of specimens and key test results.

Group	Specimen	$b_{f}$	$s_{f}^{\prime }$	$t_{f}$	$\rho_{f}$	$\rho_{f}k_{v}$	$s_{f}^{\prime }/b$	PIV	$f_{co}^{\prime }$	$\varepsilon_{co}$	$f_{cc}^{\prime }$	$\varepsilon_{cc}$	$f_{cu}^{\prime }$	$\varepsilon_{cu}$	$f_{cc}^{\prime }/f_{co}^{\prime }$	$\varepsilon_{cu}/\varepsilon_{co}$	$\varepsilon_{h,rup}$	$\varepsilon_{h,rup},\varepsilon_{f}$
	R-0	—	—	—			/	N.A.	34.74	0.0027	N.A.	N.A.	N.A.	N.A.	N.A.	N.A.	N.A.	N.A.
	F-1	N.A.	N.A.	0.167	0.33	0.33	0.00	√	34.74	0.0027	37.16	0.0037	29.70	0.0102	1.07	3.73	−0.0101	0.53
I	F-2	N.A.	N.A.	0.334	0.67	0.67	0.00	√	34.74	0.0027	46.12	0.0124	N.A.	0.0124	1.33	4.55	−0.0099	0.52
	F-3	N.A.	N.A.	0.501	1.00	1.00	0.00	√	34.74	0.0027	62.13	0.0245	N.A.	0.0245	1.79	8.98	−0.0136	0.71
	P-1-80-40	80	40	0.167	0.22	0.18	0.20	√	34.74	0.0027	34.79	0.0048	27.59	0.0089	1.01	3.25	−0.0077	0.40
II	P-2-80-40	80	40	0.334	0.45	0.36	0.20	√	34.74	0.0027	41.49	0.0039	38.26	0.0105	1.19	3.84	−0.0136	0.71
	P-3-80-40	80	40	0.501	0.67	0.54	0.20	√	34.74	0.0027	45.92	0.0201	N.A.	0.0201	1.32	7.36	−0.0134	0.70
	P-1-100-40	100	40	0.167	0.24	0.19	0.20	√	34.74	0.0027	36.25	0.0041	30.51	0.0160	1.04	5.87	−0.0161	0.84
III	P-2-100-40	100	40	0.334	0.48	0.39	0.20	√	34.74	0.0027	40.28	0.0040	38.47	0.0095	1.16	3.46	−0.0124	0.65
	P-3-100-40	100	40	0.501	0.72	0.58	0.20	√	34.74	0.0027	46.02	0.0210	N.A.	0.0210	1.32	7.68	−0.0147	0.77
	P-1-120-40	120	40	0.167	0.25	0.20	0.20	√	34.74	0.0027	37.06	0.0033	32.42	0.0082	1.07	3.00	−0.0110	0.58
IV	P-2-120-40	120	40	0.334	0.50	0.41	0.20	√	34.74	0.0027	38.87	0.0056	38.37	0.0254	1.12	9.28	−0.0151	0.79
	P-3-120-40	120	40	0.501	0.75	0.61	0.20	√	34.74	0.0027	47.53	0.0226	N.A.	0.0226	1.37	8.27	−0.0161	0.84
V	P-3-40-20	40	20	0.501	0.67	0.60	0.10	√	34.74	0.0027	47.63	0.0154	N.A.	0.0154	1.37	5.64	−0.0127	0.66
	P-3-120-60	120	60	0.501	0.67	0.48	0.30	√	34.74	0.0027	46.92	0.0057	44.10	0.0185	1.35	6.77	−0.0138	0.72
	P-3-30-20	29.8	20	0.501	0.60	0.54	0.10	√	34.74	0.0027	44.91	0.0128	N.A.	0.0128	1.29	4.68	−0.0123	0.64
	P-3-178-60	177.5	60	0.501	0.75	0.54	0.30	√	34.74	0.0027	46.52	0.0038	44.41	0.0217	1.34	7.94	−0.0121	0.63

Note: $b_{f}$ = width of FRP rings; $s_{f}^{\prime }$ = FRP strip clear spacing; $t_{f}$ = thickness of FRP rings; $\rho_{f}$ = FRP volumetric ratio; b = width of the square column cross-section; $f_{co}^{\prime }$ = compressive strength of unconfined concrete; $\varepsilon_{co}$ = axial strain of concrete at peak stress; $f_{cc}^{\prime }$ = peak stress of confined concrete; $\varepsilon_{cc}$ = axial strain of confined concrete at peak stress; $f_{cu}^{\prime }$ = ultimate stress of confined concrete; $\varepsilon_{cc}$ = axial strain of confined concrete at ultimate stress; $\varepsilon_{h,rup}$ = FRP hoop tensile rupture strain; $k_{v}$ = vertical confinement efficiency factor; $\rho_{f}k_{v}$ = FRP confinement efficiency ratio; PIV = particle image velocimetry; N.A. = not applicable.

**Table 2:** Concrete mix proportions.

Cement (kg)	Fine aggregate (kg)	Coarse aggregate (kg)	Water (kg)	Sand ratio (%)
315.4	714.3	1165.4	205.0	38.0

**Table 3:** Tensile test results of CFRP flat coupons.

Specimen	Tensile strength (MPa)		Ultimate strain (%)		Modulus of elasticity (GPa)	
	$f_{f}$	Average	$\varepsilon_{f}$	Average	$E_{f}$	Average
C-1	4222.7	4308.6	1.85	1.91	226.3	227.3
C-2	4275.4		1.85		235.2	
C-3	4419.5		1.98		217.2	
C-4	4222.7		1.90		222.9	
C-5	4402.4		1.97		235.0	

**Table 4:** Summary of existing stress–strain models for concrete in FRP-confined square columns.

Model	Ultimate Condition	Transition Point	Shape Factor	Diameter of Equivalent Circular Column (D)	FRP Strain Efficiency Factor
Lam and Teng, 2003 [29]	$\frac{f_{cc}^{\prime }}{f_{co}^{\prime }}=1.0+3.3k_{s1}\left(\frac{f_{l,a}}{f_{co}^{\prime }}\right)$ $\frac{\varepsilon_{cu}}{\varepsilon_{co}}=1.75+12k_{s2}\frac{f_{l,a}}{f_{co}^{\prime }}{\left(\frac{\varepsilon_{h,rup}}{\varepsilon_{co}}\right)}^{0.45}$	Determined by the smooth connection condition	$k_{e}=1-\frac{\left(b/h\right){\left(h-2r\right)}^{2}+\left(h/b\right){\left(b-2r\right)}^{2}}{3A_{g}\left(1-\rho_{sc}\right)}$$k_{s1}={\left(\frac{b}{h}\right)}^{2}k_{e}$, $k_{s2}={\left(\frac{h}{b}\right)}^{0.5}k_{e}$	$D=\sqrt{h^{2}+b^{2}}$	Obtained from accompanying compression tests of FRP-confined circular columns
Wei and Wu, 2012 [33]	$\frac{f_{cu}^{\prime }}{f_{co}^{\prime }}=0.5+2.7k_{s}^{0.4}{\left(\frac{f_{l,a}}{f_{co}^{\prime }}\right)}^{0.73}{\left(\frac{h}{b}\right)}^{-1}$ $\frac{\varepsilon_{cu}}{\varepsilon_{co}}=1.75+12{\left(0.36k_{z}+0.64\right)}^{0.4}{\left(\frac{f_{l,a}}{f_{co}^{\prime }}\right)}^{0.75}{\left(\frac{30}{f_{co}^{\prime }}\right)}^{0.62}{\left(\frac{h}{b}\right)}^{-0.3}$	$\frac{f_{o}}{f_{co}^{\prime }}=1.0+0.43k_{s}^{0.68}{\left(\frac{h}{b}\right)}^{-1}\left(\frac{f_{lu}}{f_{co}^{\prime }}\right)$ $\varepsilon_{0}=\frac{\left(f_{o}+f_{cu}^{\prime }+E_{c}\varepsilon_{cu}\right)-\sqrt{{\left(f_{o}+f_{cu}^{\prime }+E_{c}\varepsilon_{cu}\right)}^{2}-8f_{o}E_{c}\varepsilon_{cu}}}{2E_{c}}$	$k_{s}=2r/b$	D=b	$\varepsilon_{h,rup}=\varepsilon_{f}$
Cao et al., 2016 [54]	$\frac{f_{cu}^{\prime }}{f_{co}^{\prime }}=1+8.34{\left(\frac{2E_{f}t_{f}}{bE_{c}}\right)}^{1.03}{\left(\frac{2r_{c}}{b}\right)}^{0.81}{\left(\frac{30}{f_{co}^{\prime }}\right)}^{0.54}{\left(\frac{h}{b}\right)}^{-1.9}{\left(\frac{\varepsilon_{f}}{\varepsilon_{co}}\right)}^{0.82}$ $\frac{\varepsilon_{cu}}{\varepsilon_{co}}=1.75+9.45{\left(\frac{2E_{f}t_{f}}{bE_{c}}\right)}^{0.68}\left(0.54\frac{2r_{c}}{b}+0.46\right){\left(\frac{30}{f_{co}^{\prime }}\right)}^{0.79}{\left(\frac{h}{b}\right)}^{-0.64}{\left(\frac{\varepsilon_{f}}{\varepsilon_{co}}\right)}^{1.14}$	$\frac{f_{o}}{f_{co}^{\prime }}=1.0$ $\varepsilon_{o}={f_{co}^{\prime }b/2E_{f}t}_{f}\varepsilon_{f}$	$k_{s}=2r/b$	D=b	$\varepsilon_{h,rup}=\varepsilon_{f}$
Guo et al., 2019 [63]	$\frac{f_{cc}^{\prime }}{f_{co}^{\prime }}=1.0+2.0\left(\rho_{Ke}-0.01\right)\rho_{\varepsilon }$ $\frac{\varepsilon_{cu}}{\varepsilon_{co}}=1.75+5.5\rho_{Ke}^{0.8}\rho_{\varepsilon }^{1.45}$	Determined by the smooth connection condition	$k_{e}=1-\frac{\left(b/h\right){\left(h-2r\right)}^{2}+\left(h/b\right){\left(b-2r\right)}^{2}}{3A_{g}\left(1-\rho_{sc}\right)}$$k_{s1}={\left(\frac{b}{h}\right)}^{2}k_{e}$, $k_{s2}={\left(\frac{h}{b}\right)}^{0.5}k_{e}$	$D=\sqrt{h^{2}+b^{2}}$	Obtained from accompanying compression tests of FRP-confined circular columns

Note: $A_{g}$—gross area of the column section with rounded corners; $\rho_{sc}$—cross sectional area ratio of the longitudinal steel reinforcement; $\rho_{f}$—FRP volumetric ratio, $\rho_{\varepsilon }$—confinement strain ratio, $\rho_{Ke}$—effective confinement stiffness ratio; $r_{c}$ —corner radius; b —width of the cross section; h — length of the cross section.
